# 24 Hour ST Segment Analysis in Transient Left Ventricular Apical Ballooning

**DOI:** 10.1371/journal.pone.0058349

**Published:** 2013-03-07

**Authors:** Frank Bode, Christof Burgdorf, Heribert Schunkert, Volkhard Kurowski

**Affiliations:** Medical Department II, University of Luebeck, Luebeck, Germany; S.G.Battista Hospital, Italy

## Abstract

**Objective:**

The etiologic basis of transient left ventricular apical ballooning, a novel cardiac syndrome, is not clear. Among the proposed pathomechanisms is coronary vasospasm. Long-term ST segment analysis may detect vasospastic episodes but has not been reported.

**Methods:**

30 consecutive patients with transient left ventricular apical ballooning, left ventricular dysfunction and normal or near-normal coronary arteries were investigated. A 24-hour Holter ECG was obtained after emergency admission. ST segment analysis was performed automatically in 2 leads and confirmed by visual inspection. Criteria for an ischemic event were: 1. ST elevation or 2. horizontal or down-sloping ST segments ≥1 min duration and ≥100 µV J+80 point deviation corrected for baseline ST-deviation.

**Results:**

Patients presented with ST segment elevation (n = 19) and/or T wave inversion (n = 20) on admission ECG. Ejection fraction was 50±12%. No transient ST elevations were observed during Holter ECG analysis. In 3 patients, 8 transient episodes of ST depression were recorded. Durations of episodes varied between 75s and 790s (mean 229s). Maximal ST deviation averaged −191±71 µV. Ischemic burden was −1 to −22 mVs (mean −8 mVs). 27 patients showed no ischemic events.

**Conclusions:**

ST segment analysis of 24 h Holter recordings revealed minor ischemic events in only 10% of patients with transient left ventricular apical ballooning. Overall, ST segment changes were not indicative of recurrent coronary spasm playing a major role in the genesis of transient left ventricular apical ballooning.

## Introduction

The transient left ventricular apical ballooning syndrome, also known as takotsubo cardiomyopathy, is an acute cardiac syndrome that has only recently been generally recognized [1], [2], [3], [4], [5]. The syndrome is characterized by regional contractile dysfunction of the left ventricular apex and/or midmyocardium [6], [7] in the absence of obstructive atherosclerotic coronary disease. Regional wall-motion abnormalities extend beyond a single epicardial vascular distribution. The syndrome most frequently affects postmenopausal women immediately after an episode of acute emotional or physical stress [8], [9]. The clinical presentation is similar to that of patients with acute myocardial infarction. Patients often complain chest pain at rest or dyspnea. New electrocardiographic ST segment elevations or T wave inversions are commonly found during the acute onset of the syndrome [10]. Cardiac enzyme and biomarker levels are elevated. Acute left-sided heart failure, hemodynamic instability and arrhythmias may develop. Left ventricular dysfunction is generally reversible and overall in-hospital prognosis is favorable [8], [11]. The cause of the syndrome is unknown. Endomyocardial biopsy in the acute phase of the syndrome revealed no evidence of myocarditis [3], [12]. Spontaneous or provocable multivessel epicardial spasm was reported in a subset of patients undergoing coronary angiography, suggesting a possible role of coronary spasm in the genesis of the syndrome [1], [8], [13]. Intermittent spontaneous vasospasm can be detected in Holter ECG recordings by ST segment analysis. We evaluated the incidence of transient ischemic episodes suggestive of intermittent coronary vasospasm in the acute phase of left ventricular apical ballooning by ST segment analysis in 24 h Holter recordings.

## Methods

**Figure 1:**
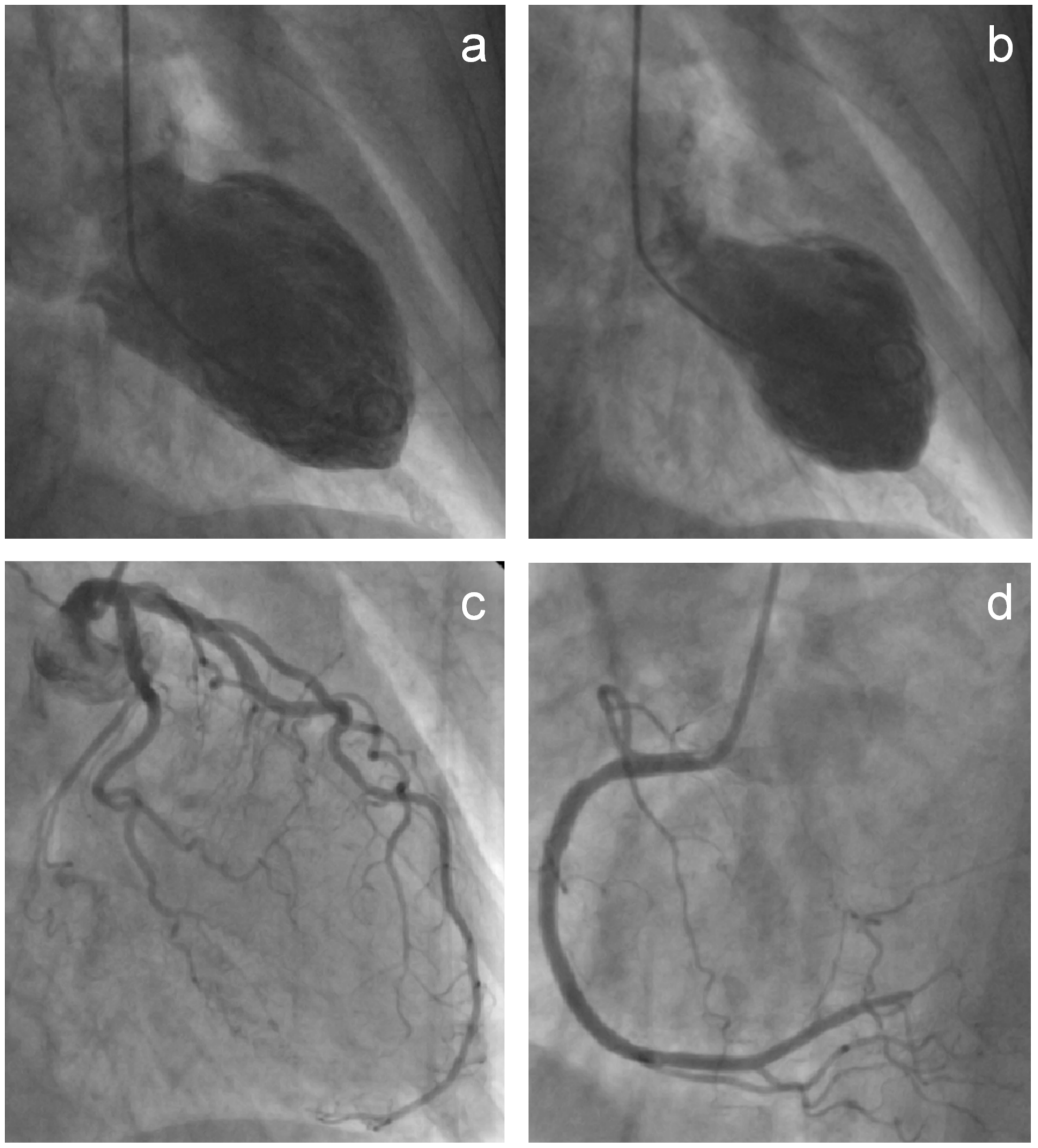
Typical left ventricular apical ballooning. Left ventriculography in right anterior oblique projection during diastole (1a) and systole (1b) demonstrating akinesia of the apical and midventricular segments. Selective coronary angiograms of the left (1c) and right coronary artery (1d) excluding obstructive coronary artery disease.

After approval by the ethics committee of the University of Luebeck, Germany, 30 consecutive patients with transient left ventricular apical ballooning syndrome were investigated. Patients were admitted to our institution for acute anginal symptoms or dyspnea within 48 hours of symptom onset. All patients presented with 12-lead ECGs suspective of acute myocardial ischemia. ECG changes were classified according to the presence of ST segment elevations ≥100 µV and/or T wave inversions. Patients underwent immediate left heart catheterization. They were included after akinesia or hypokinesia had been detected in the apical and/or midventricular region by laevocardiography while coronary angiograms ruled out obstructive coronary artery disease (Fig. 1). Cardiac marker levels were determined repetitively on admission, 6, 12 and every 24 hours after admission. Troponin T was measured quantitatively by ELISA test (ES 300 System, Roche Diagnostics) with a cut-off at 0.1 ng/ml for a positive test.

All patients underwent 24 h Holter ECG recording within two days following hospital admission after written informed consent had been obtained. Patients were instructed to protocol any symptoms during acquisition. Two bipolar leads were recorded simultaneously. The recorders used were Reynolds Medical Tracker 3 and the analyzer was a Pathfinder 600 (Reynolds Medical Limited, Hertford, England). Following analog-digital conversion of the tape recordings, the following points from the digitized ECG were used for measurements:

1. PR reference point: The midpoint of the PR segment
2. J point: The end of the QRS complex
3. J+80 measurement point: The point on the ST segment 80 ms after the J point

These points were selected from an average beat superimposition display with adjustable markers. Ischemic episodes were defined as 1. ST elevation or 2. horizontal or down-sloping ST segment depression for at least one minute with at least 100 µV J+80 measurement point deviation compared to J+80 measurement points at baseline. The baseline was defined as the mean value of PR reference points during a 5-minute period within 30 minutes prior to the episode, where baseline conditions were considered to be present based upon a relatively slow heart rate and steady ST segment. Each episode was characterized by slope of ST segment, duration, and ischemic burden (duration multiplied by deviation), and confirmed by visual inspection. Positional changes were excluded.

Variables are presented as mean±SD. Patient data were statistically compared by paired or unpaired t test for continuous variables and by Fisheŕs exact test for parametric variables. P values <0.05 defined statistical significance.

## Results

### Study Population

All 30 patients were postmenopausal women with a mean age of 71±8 years (range 56–84). Symptom onset was 12±11 hours before hospital admission. An episode of emotional stress preceded clinical presentation in 15 patients. Left ventricular ejection fraction was 50±12% during ventriculography on admission. Coronary angiographies revealed no obstructive coronary artery disease and no macrovascular spasm in any vessel. Initial and maximal troponin T serum levels were 0.72±1.08 ng/ml and 0.90±1.07 ng/ml, respectively (p = 0.002). Initial and maximal creatine kinase levels were 158±93 IU/l and 253±202 IU/l (p = 0.008), creatine kinase MB levels were 24±13 IU/l and 29±16 IU/l (p = 0.005), respectively. Patients underwent hemodynamic monitoring and received heart failure therapy as required. No nitrates or calcium channel blockers known to prevent coronary spasm were applied. Treatment with ß-blockers (n = 23) and angiotensin-converting enzyme inhibitors (n = 24) or angiotensin receptor antagonists (n = 2) was initiated within the first three days. All patients showed symptomatic and hemodynamic improvement during hospitalization for 11±6 days. Echocardiographic reevaluation before discharge showed regression of left ventricular wall motion abnormalities with an improved left ventricular ejection fraction of 61±10% (p<0.001 as compared to ventriculography on admission).

### ECG Presentation

12-lead ECGs on admission revealed no left ventricular hypertrophy or left bundle branch block that could have precluded accurate interpretation of ST segment activity. Digoxin and other medications known to affect ST segment morphology were absent. All patients were in sinus rhythm. Patients presented with ST segment elevation of at least 100 µV (n = 19) and/or T wave inversion (n = 20) on admission ECG. Patients displaying ST segment elevations on admission had a more recent symptom onset (8±8 hours) than patients without ST segment elevation (18±13 hours; p = 0.037). ST segment elevation resolved within the first two days, whereas T wave inversion resolved more slowly and often only partially.

### Holter ECG Analysis

**Figure 2:**
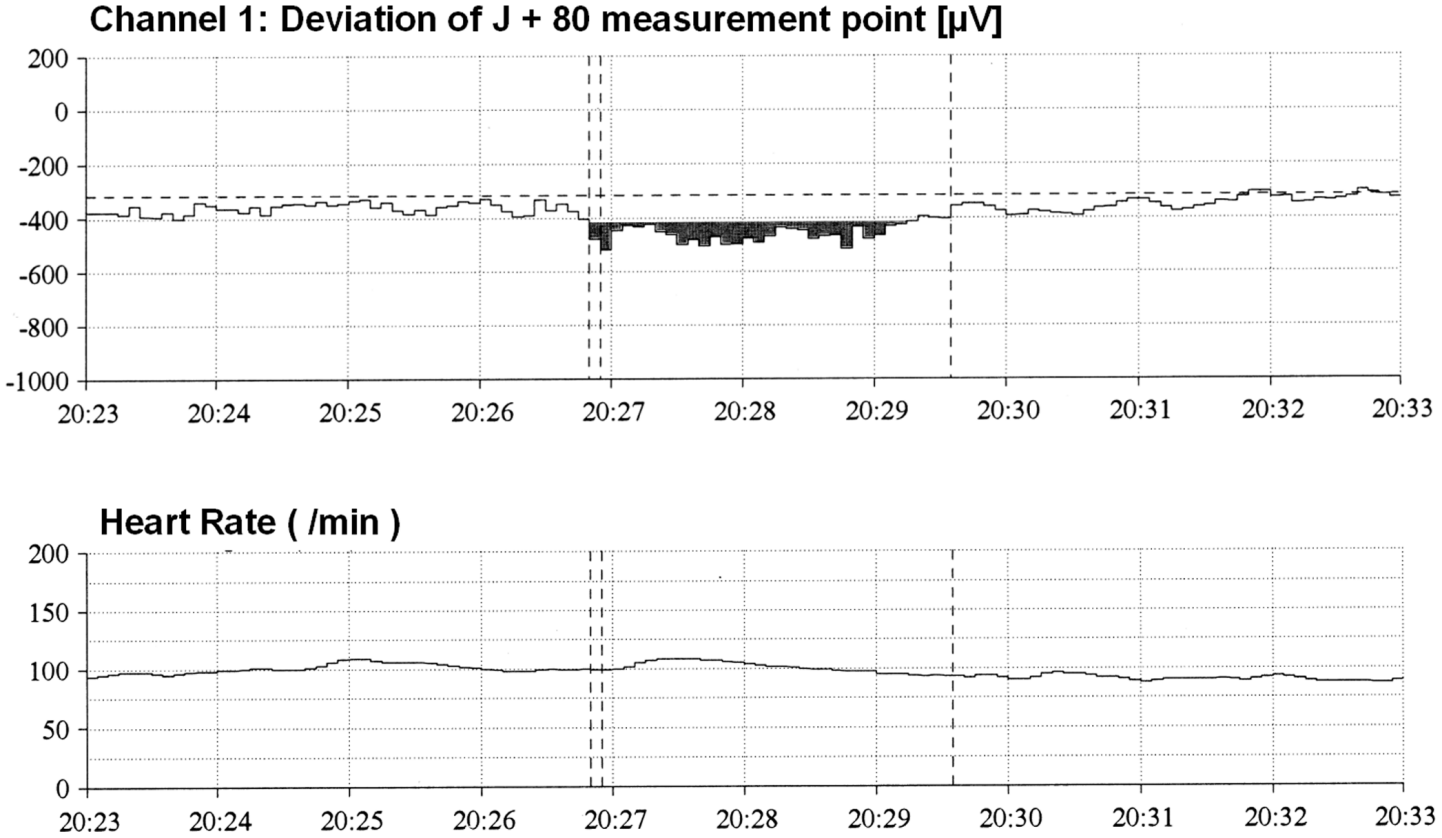
Automatic analysis of ST segment changes during Holter recording. The upper image depicts the deviation of the J +80 measurement point (80 ms after J point) within a 10 minute period (pt. no. 7). At 20 h 26 min 50 s an ischemic episode of 2 min 45s duration was detected. J +80 measurement point deviation >100 µV is marked in black. The lower image depicts the corresponding heart rate (HR).

**Figure 3:**
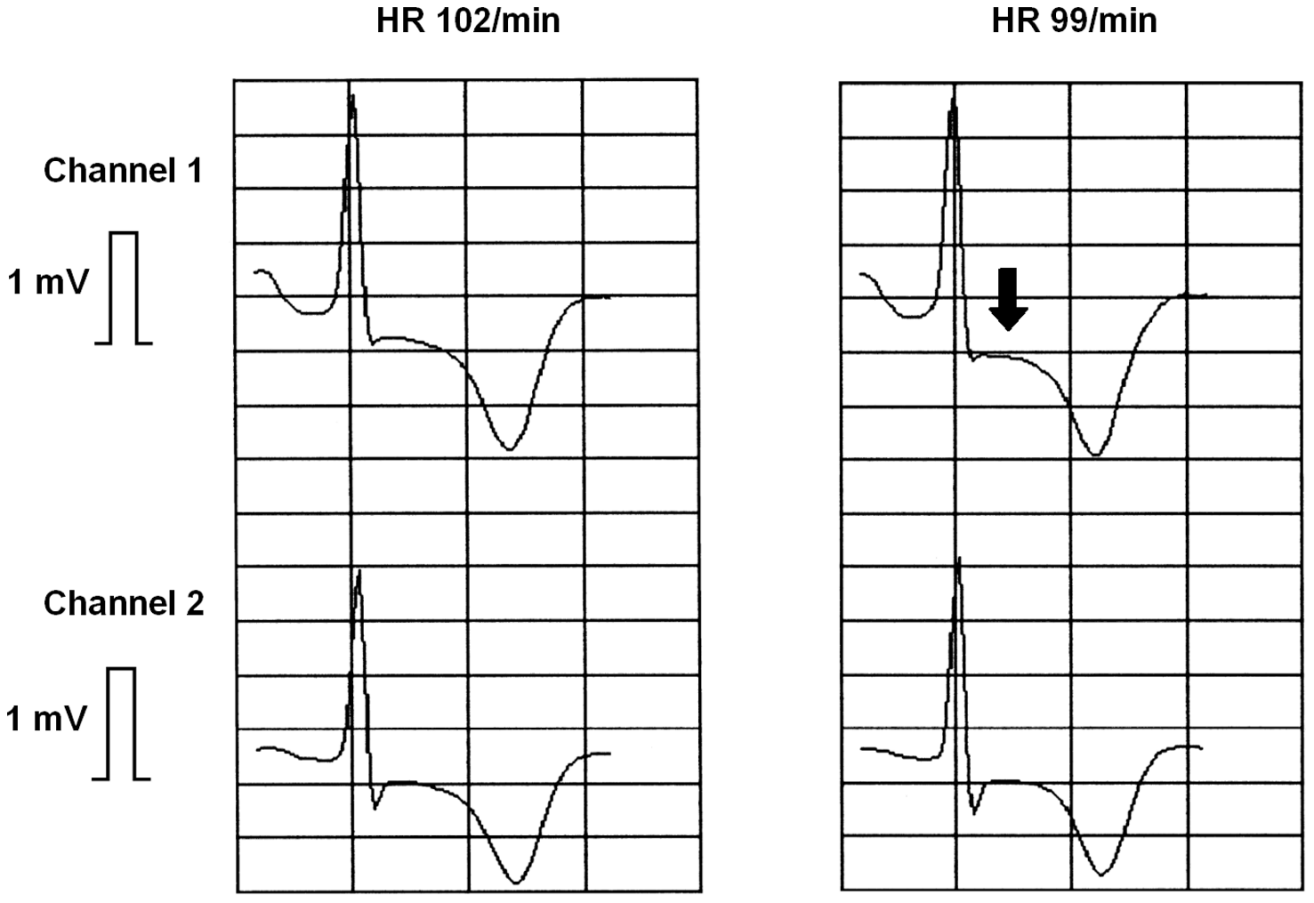
ST segment detail. Recordings show baseline ST depression in both recording channels (left tracing). During an automatically detected ischemic event at 20 h 26 min 59s an additional transient ST depression occurs in channel 1 (arrow, right tracing).

All 30 Holter recordings were of good quality. No transient ST elevations were observed during Holter ECG analysis. Positional changes mimicking transient ST depression were seen in 5 patients, none of whom had nonpositional ischemic events. In three patients, 8 nonpositional transient episodes of ST depression were recorded (Fig. 2 and 3; Table 1 ). Durations of episodes varied between 75s and 790s (mean 229s). Maximal ST deviation averaged −191±71 µV. Ischemic burden was −1 to −22 mVs (mean −8 mVs). Heart rate was 74±19 bpm before and 77±19 bpm during episodes (p = 0.718). No patient reported clinical symptoms during automatically detected episodes of ST depression.

**Table 1:** Ischemic events.

Patient	Event	Time	Duration	Heart Rate before event	Heart Rate during event	Max. Deviation	Ischemic Burden
No.		(h:min:s)	(min:s)	(bpm)	(bpm)	(µV)	(mVs)
1	ST depression	00∶54:15	02∶10	55	56	−138	−2
1	ST depression	14∶02:20	01∶15	56	55	−132	−1
1	ST depression	18∶11:45	03∶40	60	55	−124	−1
7	ST depression	12∶15:00	13∶10	86	89	−209	−22
7	ST depression	12∶55:00	01∶30	83	90	−202	−3
7	ST depression	13∶57:05	04∶05	89	91	−173	−3
7	ST depression	20∶26:50	02∶45	102	99	−201	−7
16	ST depression	12∶46:20	02∶00	57	81	−345	−22

### Characteristics of Patients with Ischemic Events

The three patients with transient ST segment depression displayed T wave inversion but no ST segment elevation on admission ECG. Maximal cardiac marker levels were lower in those patients than in the remaining study population (maximal troponin T level 0.19±0.13 ng/ml vs. 0.98±1.10 ng/ml; p = 0.015) but not lower than in the other patients without ST segment elevation (0.21±0.16; p = 0.823). Apart from this observation, patients with transient episodes of ST depression showed no specifically different clinical features or history from the 27 patients which showed no ischemic events.

## Discussion

The present study investigated for the first time the incidence of transient ischemic episodes in the acute phase of transient left ventricular apical ballooning syndrome by 24 h Holter recordings. ST segment analysis revealed only few, brief ischemic episodes in a minority of patients. Our findings were not indicative of recurrent coronary spasm forming a crucial substrate of this novel cardiac syndrome.

### Relevance of Ischemic Episodes

ST segment analysis by Holter monitoring is an established method for detection of intermittent ST deviations indicative of ischemia. A good correlation has been established between ischemic events during exercise tests and ST segment changes during Holter Monitoring [14], [15]. Holter monitoring has facilitated the assessment of silent ischemia in different clinical settings [16], [17]. Due to the transient nature of coronary vasospasm, Holter Monitoring appears to be the proper method for detecting brief spontaneous episodes of resulting ischemia. ST segment analysis by Holter monitoring is indicated for detection of vasospastic angina [18].

The 10% of our patients which displayed transient ischemic events in Holter recordings belonged to a subgroup of patients without ST segment elevation during initial ECG presentation and with a comparatively small release of cardiac markers on admission. Moreover, the brief ischemic events that were detected during Holter recordings caused no symptoms and triggered no further rise in cardiac markers. In contrast, in patients with ST segment elevation on admission reaching high peak plasma levels of cardiac markers, Holter analysis found no transient ischemic events. Thus, electrical and laboratory correlates of myocardial damage were sparse in patients with transient ischemic events. These findings argue against recurrent episodes of ischemia as a relevant pathomechanism driving the syndrome.

### Pathology of the Syndrome

Originally reported in the Japanese population [1], [13], the transient left ventricular apical ballooning syndrome has recently been described in European and U.S. patients [19], [20]. Most patients meet the criteria for the diagnosis of myocardial infarction upon presentation, but obstructive epicardial coronary disease is absent. Some features of the syndrome are similar to those seen in myocarditis, but no evidence of myocarditis could be demonstrated in endomyocardial biopsies or viral serology [3], [10], [12]. Regional perfusion abnormalities have been documented by coronary flow measurements and SPECT imaging, which were reversible after the acute phase of the syndrome. Beyond regional perfusion abnormalities, fatty acid and glucose metabolism was found to be impaired in affected regions, indicative of the presence of myocardial “stunning” [21], [22], [23], [24]. Several mechanisms have been proposed to underlie the transient left ventricular apical ballooning syndrome, including multivessel epicardial spasm [10], microvascular coronary spasm [3], [13] and catecholamine-mediated myocardial dysfunction [12], [25].

### Proposed Mechanisms

Patients with transient left ventricular apical ballooning syndrome often present in the setting of acute mental or physical stress, associated with ***enhanced sympathetic outflow*** [5], [12], [26]. It remains unproven whether elevated catecholamine levels represent the causal factor of the syndrome or an epiphenomenon secondary to hemodynamic abnormalities. On one hand, plasma catecholamine levels were higher in transient left ventricular apical ballooning syndrome than in control patients with comparable hemodynamic compromise due to acute myocardial infarction [12]. In a rat model of emotional stress, regional wall motion abnormalities could be reproduced while α- and ß-blocker pretreatment abolished those changes [27]. These findings supported a causal role of sympathetic stimulation. On the other hand, analysis of heart rate variability and turbulence, surrogate markers of autonomic tone, showed that the cardiac sympathetic modulation was not elevated in transient left ventricular apical ballooning syndrome, but sympathovagal balance was rather preserved [28].

***Transient epicardial spasm*** would be required to occur in multiple vessels in order to cause the diffuse wall motion abnormalities located beyond a single epicardial distribution. Spontaneous multivessel spasm has been observed in up to 6% of patients in single centers [13]. A recent meta-analysis of 212 patients reported an overall incidence of only 1.4% [8]. Provocable coronary vasospasm in at least one coronary artery has been found in 10/14 (71%) [13], 10/48 (21%) [2], 2/5 (40%) [1] and 1/7 (14%) [3] of patients. Multivessel spasm could be provoked by ergonovine or acetylcholine in 29% of 84 evaluated patients [8]. Multivessel epicardial spasm might therefore play a role in some patients presenting with the syndrome. Yet, in patients with persistent ST elevations and no evidence of epicardial spasm during coronary angiography (19 of 30 patients in our study population), this genesis is not plausible.

While macrovascular changes are rarely observed, ***microvascular dysfunction*** could be responsible for inducing myocardial ischemia and subsequent myocardial stunning. Microvascular function has been assessed by invasive measurements of coronary flow reserve [29], [30], [31] and TIMI frame count techniques [13], [23], [32]. The vast majority of data supported the hypothesis that abnormal microcirculation contributes to the pathophysiology of the syndrome. Coronary flow reserve was impaired and TIMI myocardial perfusion grade was reduced in most patients. The severity of myocardial injury as measured by peak troponin levels and the presence of ST elevation has been correlated to the impairment of myocardial perfusion [32]. It remains unclear whether microvascular dysfunction is the primary cause of the syndrome or a secondary phenomenon. Abnormal microcirculation might be secondary to the cardiomyopathy and elevated LV filling pressures. But this has been refuted by the assessment of normal microcirculation in patients without the syndrome who showed similar degrees of LV dysfunction and filling pressures [32].

***Microvascular coronary spasm*** might be operative as primary cause of microvascular dysfunction. Nicorandil, a K_ATP_ channel opener that causes vasodilatation of arterioles and large coronary arteries, reduced the extend of ST segment elevation when injected into the coronary arteries, suggesting that microvascular spasm may underly abnormal microcirculation [33]. While the long-term clinical history of each of our patients was devoid of episodes of chest discomfort or variant angina, in particular, and thus revealed no overt disposition for coronary spasms, alterations in vasomotor activity might have been acquired. The predominance of postmenopausal women in patients presenting with the syndrome has suggested that changes in sex hormone activity are involved in its development. Reduced estrogen levels may alter endothelial function and microcirculatory vasomotor activity in response to catecholamines [34], [35], [36], [37]. Estradiol supplementation in ovariectomized female rats attenuated stress-induced wall-motion abnormalities [34].

An increased susceptibility to repetitive coronary spasm was not supported by the results of our study. Few patients showed minor transient ST segment changes with small ischemic burden during the acute phase of the syndrome, while in 90% of patients Holter ECG recording revealed no transient ischemia. Therefore microvascular dysfunction could not be attributed to recurrent coronary spasm in the majority of our patients. Only few patients who have suffered from a first apical ballooning episode experience a repeat episode. This clinical course might further argue against a persistent susceptibility to coronary spasm, thereby supporting our results.

It has to be considered that microcirculatory impairment during the acute phase is not causative to the syndrome but could result from primary myocardial injury. The syndrome might speculatively represent catecholamine-mediated toxicity and metabolic injury rendering the heart to diffuse microvascular dysfunction and myocardial stunning [4], [25], [38]. Opposed to this, Angelini postulated that a catecholamine surge by itself would be insufficient to explain the occurrence of the syndrome [39]. Due to the documentation of provocable diffuse spasm of all coronary distal branches in patients recovering from apical ballooning, his pathophysiologic theory regards the catecholamine surge only as a preciptating factor in labile, endothelially dysfunctional patients. The liability to extreme spasticity is postulated to be transient, varying from a few hours to a few weeks [39]. Our Holter recordings were obtained within 48 h after hospital admission. Without using provocation tests, we found no relevant indication of recurrent coronary spasms in this early phase after manifestation. An unusual spasticity was not supported by our findings. Thus, only in rare cases a persistent susceptibility to coronary spasm might contribute to the development of transient left ventricular apical ballooning syndrome.

### Limitations

Holter ECG recordings were obtained within 48 h after hospital admission. Due to the dynamic nature of transient left ventricular ballooning with acute onset and short-term recovery, the Holter recording might not reflect the initial susceptibility to coronary vasospasm. A spasm preceding the onset of an apical ballooning episode cannot be excluded by any method applied after clinical presentation. We did not use pharmacologic provocation to test the susceptibility to coronary spasms in our patients and to correlate them with the Holter results. Yet, the low incidence of brief spontaneous ischemic episodes during Holter evaluation argues against the usefulness of provocation tests to detect clinically relevant vasospastic instability.

### Conclusions

While the pathophysiological roles of multivessel epicardial spasm, microvascular coronary spasm and catecholamine-mediated myocardial dysfunction in transient left ventricular apical ballooning syndrome are still under debate, ST segment analysis during the acute phase of the syndrome provided further evidence against repetitive vasospasm as a relevant contributor to the genesis of the syndrome. Microvascular dysfunction could not be attributed to recurrent vascular spasms in the majority of patients.
